# Differential expression patterns of long noncoding RNAs in a pleiomorphic diatom and relation to hyposalinity

**DOI:** 10.1038/s41598-023-29489-w

**Published:** 2023-02-10

**Authors:** Ahmed Debit, Florent Charton, Priscillia Pierre-Elies, Chris Bowler, Helena Cruz de Carvalho

**Affiliations:** 1 Institut de Biologie de L’ENS (IBENS), Département de Biologie, École Normale Supérieure, CNRS, INSERM, Université PSL, 75005 Paris, France; 2 Faculté des Sciences et Technologie, Université Paris Est-Créteil (UPEC), 61, Avenue du Général De Gaulle, 94000 Créteil, France

**Keywords:** Plant sciences, Plant cell biology, Plant molecular biology

## Abstract

Long non-coding (lnc)RNAs have been shown to have central roles in stress responses, cell identity and developmental processes in multicellular organisms as well as in unicellular fungi. Previous works have shown the occurrence of lncRNAs in diatoms, namely in *Phaeodactylum tricornutum*, many of which being expressed under specific stress conditions. Interestingly, *P. tricornutum* is the only known diatom that has a demonstrated morphological plasticity, occurring in three distinct morphotypes: fusiform, triradiate and oval. Although the morphotypes are interchangeable, the fusiform is the dominant one while both the triradiate and the oval forms are less common, the latter often being associated with stress conditions such as low salinity and solid culture media, amongst others. Nonetheless, the molecular basis underpinning morphotype identity in *P. tricornutum* remains elusive. Using twelve previously published transcriptomic datasets originating from the three morphotypes of *P. tricornutum*, we sought to investigate the expression patterns of lncRNAs (lincRNAs and NATs) in these distinct morphotypes, using pairwise comparisons, in order to explore the putative involvement of these noncoding molecules in morphotype identity. We found that differentially expressed lncRNAs cluster according to morphotype, indicating that lncRNAs are not randomly expressed, but rather seem to provide a specific (noncoding) transcriptomic signature of the morphotype. We also present evidence to suggest that the major differences in DE genes (both noncoding and coding) between the stress related oval morphotype and the most common fusiform morphotype could be due, to a large extent, to the hyposaline culture conditions rather than to the morphotype itself. However, several lncRNAs associated to each one of the three morphotypes were identified, which could have a potential role in morphotype (or cell) identity in *P. tricornutum*, similar to what has been found in both animals and plant development.

## Introduction

Diatoms are single-celled photosynthetic eukaryotes and one of the major phytoplankton groups dominating the global ocean, thus playing a central role in marine primary productivity. They are believed to have been around 200 My, the first fossils dating from the Jurassic^1,2^, having therefore survived the last recorded mass extinction on Earth, which led to the disappearance of the dinosaurs, around 66 My ago. Diatoms have a complex evolutionary history having emerged from a secondary endosymbiosis, and being subject to various horizontal gene transfers from diverse origins, attested by their chimeric genomes^3,4^. Diatoms present an intricately complex genomic content which confers them a unique combination of physiological properties. For instances they are photosynthetic organisms, like all land plants and green algae, while also presenting a fully functional urea cycle, typically found in metazoans^5^. This makes diatoms a very interesting model to study the molecular basis of a multitude of cellular processes, with the potential to transpose the knowledge to other organisms.

Long noncoding (lnc)RNAs are transcripts longer than 200 nucleotides, with low to no protein coding potential^6–8^. Like mRNAs, most lncRNAs are transcribed by the RNA polymerase II, being capped and polyadenylated and even spliced^9,10^. However, unlike mRNAs, these transcripts have been shown to be functional as RNA molecules, without the need to pass through the translation machinery. Due to the vast heterogeneity and low sequence conservation of lncRNAs, the general consensus is to classify them according to the location and orientation of the genomic location from which they are transcribed in relation to protein coding loci^10,11^. For instances, lncRNAs originating between two protein coding loci are termed intergenic (linc)RNAs and those arising from a locus which overlaps in the opposite strand with a protein coding locus are termed natural antisense transcripts (lncNATs or simply NATS). LncRNAs have been shown to regulate gene expression at multiple levels through their intrinsic capacity to interact with proteins, DNA and other RNAs^12^. This leads to a diversity of regulatory mechanisms involving changes in the chromatin landscape, the modulation of the expression of neighboring or distant genes, RNA maturation and stability and even nuclear architecture^10,12^. LncRNAs have been shown to regulate many biological processes, namely cellular differentiation, development and stress responses^10,12–17^. The majority of functional studies regarding lncRNAs stem from mammalian systems^18^ with over two thousand reports validating lncRNAs’ function in cell and developmental biology^12^. However, lncRNAs are not exclusive to mammals, nor are they to vertebrates alone. Hundreds of thousands of lncRNAs have been identified across the Eukarya domain of life, from unicellular protists and yeasts, to plants to animals^9,19–21^. In diatoms a few studies have shown the occurrence of lncRNAs^22–26^. In the model marine diatom *Phaeodactylum tricornutum*, several lncRNAs (both lincRNAs and NATs) have been shown to be expressed under specific environmental stresses like phosphate and nitrogen depletion, temperature variations and atmospheric CO_2_ pressure variation^22–26^. Additionally, several thousands of lncRNA have recently been identified in a benthic diatom (*Seminavis robusta*), which show not only a specific expression in response to environmental stresses but also a diurnal responsiveness (rhythmic activation according to a day/night cycle)^25^.

*P. tricornutum* is a raphid pennate diatom with the particularity of being only partially silicified, which seems to confer on it its morphological plasticity^27,28^. Its pleiomorphy is characterized by three well known interchangeable morphotypes: fusiform, triradiate and oval. The fusiform is the most common and abundant morphotype, representing > 95% of the morphotypes of most Pt strains, with only a few exceptions^28,29^. Cultures of the fusiform morphotype will sometimes present a small fraction of spontaneously appearing triradiate forms (1–5%). The oval morphotype is preferably a benthic form^28,30^ and interestingly, it is the only form which presents a raphe^29,31^. This morphotype is generally associated with unfavorable culture conditions (light, salinity, nutrients) or when cultured on solid media^28,29,31,32^. Ovide and collaborators have isolated and maintained in culture the three morphotypes of *P. tricornutum* using the Pt3 strain from which they characterized at the transcriptomic level, the differential expression of the annotated protein coding genes (mRNAs)^33^ leaving the non-protein coding fraction of the transcriptomes uncharacterized. We sought to fill in this gap of knowledge and analyze the expression patterns of long noncoding RNAs (lncRNAs) in the same transcriptomic datasets.

In the present study we aimed to analyze the differential expression patterns of lncRNAs (lincRNAs and NATs) between the transcriptomes of the three morphotypes of *P. tricornutum* originating from the work of Ovide and collaborators^33^, in order to provide insights into their putative involvement in morphotype (or cell) identity. Since the oval morphotype used in the aforementioned work was cultivated under lower salinity conditions than the other two morphotypes (fusiform and triradiate), in order to evaluate the salinity vs morphotype effect, we sought to compare the results obtained by the transcriptomic data with molecular expression data generated with cultures of the most common morphotype (fusiform), cultivated under normal salinity versus hyposaline conditions.

## Materials and methods

### Data sources

The raw data analyzed in this paper (Supplementary Table S1) was retrieved from the European Nucleotide Archive ENA using the accession number PRJEB26173. The data consists of strand-specific paired-end RNA-seq reads which originated from *Phaeodactylum tricornutum* (Pt3 strain) cultures of the three distinct morphotypes (fusiform, triradiate and oval)^33^. In total we analyzed four biological replicates per morphotype leading to a total of twelve paired-end samples.

### Bioinformatic analysis

The bioinformatic pipeline (Supplementary Fig. S1) was applied on the raw reads of the three morphotypes. Briefly, raw reads were processed with FASTQC (v0.11.5) to check the quality of the bases (Supplementary Fig. S2; Table S1). The strandedness of the libraries (Supplementary Table S1) was performed using the infer_expriment.py script included in the RSeQC package (v2.6.4). For the cleaning step, a home procedure in bash scripting was implemented including adapter trimming, low quality, and short reads removing (cutadapt v.3.2, and bbduk.sh script of the bbmap tool v.38.90), and removing ribosomal RNAs using sortmeRNA (v.2.1). The cleaned reads were then aligned to the *P. tricornutum* reference genome sequence (.fasta), and the reference annotation (.gtf) (v.2.47) using TopHat2 (v2.1.1) with the library strand option (–library-type fr-firststrand). The mapped reads were processed and only uniquely mapped reads were kept for the next step. The resulting files were then sorted and indexed using the samtools software suite (v.1.4.1). Read count assignment was performed with the HTSeq framework (v.0.11.3) including the strandedness information (–stranded = reverse). To measure the expression levels of long non-coding (lncRNAs) and coding (mRNAs) transcripts within the three transcriptomes (fusiform, oval and triradiate), the sets of coding and non-coding were first filtered (filtering out lowly expressed transcripts) using the CPM method from the NOISeq package. The data were then successfully normalized using the rlog function from the DESeq2 package (Supplementary Fig. S3).

### Identification of lincRNA transcripts

To identify the set of lincRNA transcripts differentially expressed between two morphotypes for each pairwise comparison, a set of 1,510 lincRNAs from *P. tricornutum*, identified and characterized in a previous study^22^, was inputted to the step 1 and step 2 of the pipeline (Supplementary Fig. S1). In step 1, the reads were summarized per lincRNAs per replicate. During step 2, the matrix of read counts was filtered for lowly expressed lincRNAs using the CPM method from the NOISeq package (v2.38.0) prior to differential expression analysis. A total of 1,426 lincRNAs were kept for downstream analysis. The read counts were then normalized using the median of ratio method of DESeq2 (v1.34.0) which corrects for sequencing depth and RNA composition biases. The differential expression of lincRNAs between morphotypes was performed using the Wald test of the DESeq function. The adjusted p-value was obtained by the Benjamini–Hochberg method, and lincRNAs with a* p*-value < 0.05 were considered as significantly differentially expressed.

### Detection and abundance of NAT-mRNA pairs

To detect NAT-mRNA pairs, the pipeline (steps 1, 2, 3; Supplementary Fig. S1) was supplied with a set of 3279 NATs from *P. tricornutum* identified and characterized in a previous study^23^. A differential expression analysis was performed for each pairwise comparison, and only DE NATs were considered. From this list, a set of all mRNA genes that overlapped with at least one NATs (overlapped region ≥ 50 bp) was identified. An annotation file (GTF) containing the positions of the mRNA genes on the genome was generated (mRNA.gtf). A read counting per mRNA per sample was performed, and a matrix of counts was produced. The matrix was then used to perform a differential expression to detect DE mRNA genes. The Pearson correlation between the DE NATs and their overlapped DE mRNA genes was calculated. All the pairs with a PCC correlation (R^2^ ≥ 0.6) were considered as putative NAT-mRNA pairs. According to the overlap pattern between the NAT and the corresponding mRNA gene, the NAT-mRNA pairs were categorized into: head-to-head (5ʹ ends overlapped), tail-to-tail (3ʹ ends overlapped), and fully overlapped. According to the direction of the correlation between the NAT and the corresponding mRNA gene, the pairs were classified as concordant and discordant. For concordant NAT pairs, both sense and antisense transcripts should change their expression level in the same direction, and for discordant NAT pairs they should change in the opposite direction.

### GO terms associated with DE NAT-mRNA pairs

To perform the Gene Ontology (GO) enrichment, a list of GO terms was obtained from the EnsemblProtists biomart portal (URL: https://protists.ensembl.org/index.html) for *Phaeodactylum tricornutum* species. The list contains the mapping between the *Gene stable ID* and *GO term accession*. For each significantly differentially expressed mRNA, GO enrichment was performed to find GO terms of the Biological Process (BP), Cellular Component (CC), and Molecular Function (MF) categories enrichment using the topGO package (v2.46.0). To measure the significance of GO terms, the Kolmogorrov-Smirnov test was used.

### Diatom culture conditions

Axenic cultures of *P. tricornutum* strain Pt1 (CCMP632) were obtained from the Center for the Culture of Marine Phytoplankton (Maine, USA). They were cultured in 50 ml f/2 media^34^ filtered steam-sterilized artificial seawater (40 g/L, NutriSelect® Basic, Merck Millipore) supplemented with f/2 nutrients, elements and vitamins (f/2 Media Kit, Bigelow) with the exception of silica, in tissue culture flask (75 cm^3^) vented filter cap (CytoOne, Starlab) under continuous shaking (90–100 rpm) at 19 °C under cool white fluorescent lights at ~ 60 µmol m^−2^ s^−1^ with a 12 h photoperiod. Culture growth was followed using a hemocytometer (Fisher Scientific, USA). Cells were harvested at the same time of day (8 h after beginning of the light period) by vacuum filtration on 0.45 µm Cytiva Nylon Membranes (Cytiva 7404-004, Fisher Scientific, France), flash-frozen in liquid nitrogen and maintained at − 80 °C until used. All the experiments were repeated at least 3 times. For the hyposalinity experiment, CCMP632 strains were cultured in 50 ml f/2 media with filtered steam-sterilized artificial sea water lowered to 10% (4 g/L, same reference as above).

### Quantitative real-time reverse transcription PCR

Total RNA was extracted from flash frozen *P. tricornutum* Pt1 cell pellets from 400 ml cultures in f/2 media containing both 40 g/L (control) and 4 g/L (hyposalinity) sea salts, using the Trizol method according to the manufacturer’s instructions (Invitrogen). Extracted RNA was treated and purified with TURBO DNA-free™ Kit (ThermoFischer Scientific) according to the manufacturer’s instructions (“routine DNase treatment”). To eliminate the DNAse treatment buffer, we included an RNA precipitation step in pure cold ethanol (2–3 v/v) with the addition of Sodium Acetate (0.1 v/v), followed by a wash step using 75% cold ethanol. The quality of the purified RNA was assessed using the Nanodrop ND1000/NanoDrop One W. For cDNA synthesis, 500 ng RNA was incubated with SuperScript III Reverse Transcriptase (Invitrogen) according to the manufacturer’s instructions (using oligo dT as primers). For quantitative RT-PCR analysis, cDNA was amplified using Takyon™ No ROX SYBR 2X MasterMix blue dTTP (Eurogentec) according to the manufacturer’s instruction with specific primers (Supplementary Table S2). Primers were designed with the Primer-Blast program (http://www.ncbi.nlm.nih.gov/tools/primer-blast/) defining PCR amplicon size between 90 and 150 bp and *Phaeodactylum tricornutum* (taxid: 2850) as the reference organism to check for primer pair specificity. Quantitative 2-step PCR conditions was set up in a Roche LightCycler 480 in a 384 well plate (Roche) under the following conditions: initial denaturation at 95 °C for 10 m followed by 40 cycles of denaturation at 95 °C for 10 s and annealing/elongation 60 °C for 40 s. Melting curve analysis was performed between 60° and 95° (ramp rate 0.04 °C/s—15 acquisitions per °C). Standard curve was generated to access primer efficiency and data were normalized using the delta-delta Ct method with Phatr3_J29812 (Actin1) and Phatr3_J19761 (tRNA ligase) mRNA levels as references.

## Results

### LncRNAs vs. mRNAs expression levels in the three morphotypes of *P. tricornutum*

**Figure 1:**
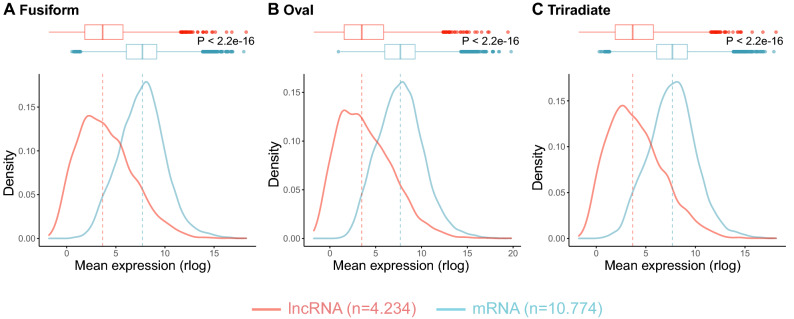
Expression level differences between coding and noncoding transcripts in *P. tricornutum* morphotypes. We measured the expression level differences between mRNAs and lncRNAs within the three transcriptomes: (**A**) Fusiform, (**B**) Oval, and (**C**) Triradiate. The sets of coding and non-coding were first filtered (filtering out lowly expressed transcripts) using the CPM method from the NOISeq package, and the number of transcripts retained for downstream analysis is displayed in parentheses. The data were then normalized using the rlog function from the DESeq2 package. Figures were made using the “Wes Anderson” color palette “Zissou1” in R (https://github.com/karthik/wesanderson).

Using the four transcriptome replicates of the three morphotypes (fusiform, oval and triradiate)^33^ (PRJEB26173), we quantified and compared the expression levels of a portfolio of previously identified lncRNAs^22,23^ and mRNAs (Phatr2/Phatr3) of *P. tricornutum* within the twelve transcriptomes. The sets of coding and non-coding RNAs were first filtered then normalized (Methods). Among the 4,234 lncRNAs (lincRNAs and NATs) and the 10,774 mRNAs analyzed, lncRNAs consistently showed statistically significant lower expression levels than mRNAs, across all transcriptomic datasets originating from the three morphotypes (Fig. 1).

### Detection of differentially expressed long noncoding RNAs (lincRNAs and NATs) between the three morphotypes of *P. tricornutum*

**Figure 2:**
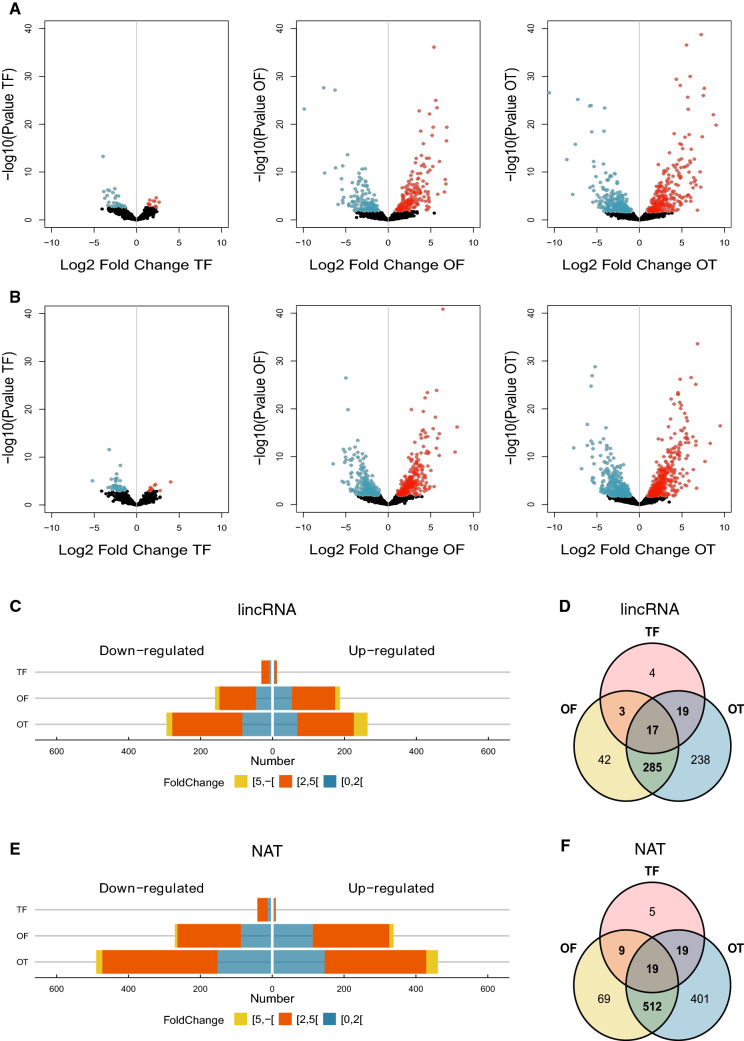
Differentially expressed long noncoding RNAs (lincRNAs and NATs) between the three morphotypes of *P. tricornutum*. Volcano plot showing the summary of the DE analysis for the three pairwise comparisons of lincRNAs (**A**) and NATs (**B**). The blue dots represent down-regulated transcripts while the red ones represent up-regulated transcripts. Dark dots represent the non-significantly DE transcripts. Pyramid plots representing the distribution of the significantly differentially expressed lincRNAs (**C**) and NATs (**E**) according to their log2FoldChange for each pairwise comparison. Venn diagram showing the intersection of DE lincRNAs (**D**) and (**F**) NATs between the three pairwise comparisons. TF, triradiate vs. fusiforme; OF, oval vs. fusiforme; OT, oval vs. triradiate. Figures were made using the “Wes Anderson” color palette “Zissou1” in R (https://github.com/karthik/wesanderson).

A pairwise comparison of the differentially expressed (DE) long noncoding RNAs (lincRNAs and NATs) in the three *P. tricornutum* morphotype cultures was performed, as was previously done for mRNAs^33^, using the available RNA-Sequencing data (PRJEB26173). The fusiform (F) morphotype was used as the reference, since it is the most common and abundant form of this diatom^28,29,33^. The three pairwise comparisons showed distinct results (Fig. 2; Supplementary Table S3) and, among the three morphotypes, the lower number of differentially expressed (DE) lncRNAs (for both lincRNAs and NATs) was found between the triradiate morphotype (T) and the fusiform (F) (noted as TF), with a strikingly larger number of DE lncRNAs between the oval (O) and the fusiform morphotypes (OF), and between the oval (O) and the triradiate (T), (noted as OT), as can be seen in the volcano plots (Fig. 2A,B). In the TF pairwise comparison we found 43 DE lincRNAs and 52 DE NATs while in the OF pairwise we found 347 DE lincRNAs and 609 DE NATs and in the OT pairwise we found 559 lincRNAs and 951 NATs DE (Fig. 2C–F; supplementary Table S3). Amongst the DE lincRNAs and NATs, 85% (302 out of 347) and 87% (531 out of 609), respectively, in the OF pairwise comparison were also DE in the OT pairwise comparison. This reveals that the expression profiles of the fusiform and triradiate morphotypes, when compared to the oval transcriptome, share the same response on 85–87% of the lncRNAs that are differentially expressed. Regarding DE genes, in the OF pairwise comparison, 187 lincRNAs (54%) and 338 NATs (56%) were upregulated, whereas 160 lincRNAs (46%) and 271 NATs (44%) were downregulated (Fig. 2C–F; Supplementary Table S3). In the OT pairwise comparison, 264 lincRNAs (47%) and 461 NATs (48%) were upregulated whereas 295 lincRNAs (53%) and 490 NATs (52%) were downregulated (Fig. 2C–F; Supplementary Table S3). In the TF pairwise comparison, 12 lincRNAs (28%) and 11 NATs (21%) were upregulated whereas 31 lincRNAs (72%) and 42 NATs (79%) were downregulated (Fig. 2C–F; Supplementary Table S3).

### Clustering of DE genes (noncoding and coding) according to morphotype

**Figure 3:**
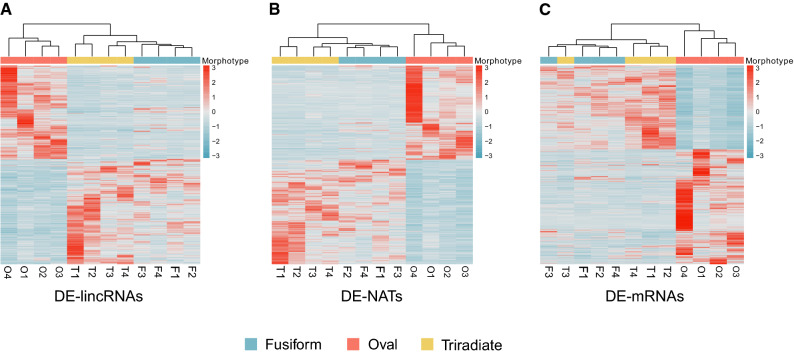
Clustering heatmaps of long noncoding (lincRNA and NAT) and coding transcripts from all three pairwise comparisons (TF, OF, OT). Heatmaps showing multi-group comparison of differentially expressed (**A**) lincRNAs (**B**) NATs, and (**C**) protein-coding transcripts (mRNA) in the three morphotypes. For each heatmap, the set of DE transcripts was taken as the union of DE transcripts of the three pairwise comparisons: T versus F, O versus F, and O versus T. DE transcripts are grouped by hierarchical clustering using the “complete” method and the “Euclidean” distance metric. The heatmaps were produced using the *pheatmap* R package. Figures were made using the “Wes Anderson” color palette “Zissou1” in R (https://github.com/karthik/wesanderson).

We sought to compare the specificity of the DE genes between the three different morphotypes, both noncoding (lincRNAs and NATs) and protein coding (mRNAs), detected in all three pairwise comparisons (TF, OF and OT). A hierarchical clustering analysis showed that the oval morphotype had a distinct DE gene signature, for both coding and noncoding genes, from the two other morphotypes (both fusiform and triradiate) in all four replicates (Fig. 3). When looking at protein coding DE genes (mRNAs), the fusiform and triradiate morphotypes do not clearly cluster separately (Fig. 3C) therefore no distinguishable “signature” of these two morphotypes could be detected. However, when analyzing lncRNAs, both lincRNAs and NATs cluster separately with each morphotype, although more stringently with three of the four replicates (Fig. 3A,B). DE lncRNAs appear to provide a more specific signature than mRNAs to the fusiform and triradiate, the two seemingly most closely related morphotypes at the molecular level (Fig. 3).

### Identification and characterization of DE NAT-mRNA pairs between the three pairwise comparisons (TF, OF, OT)

**Figure 4:**
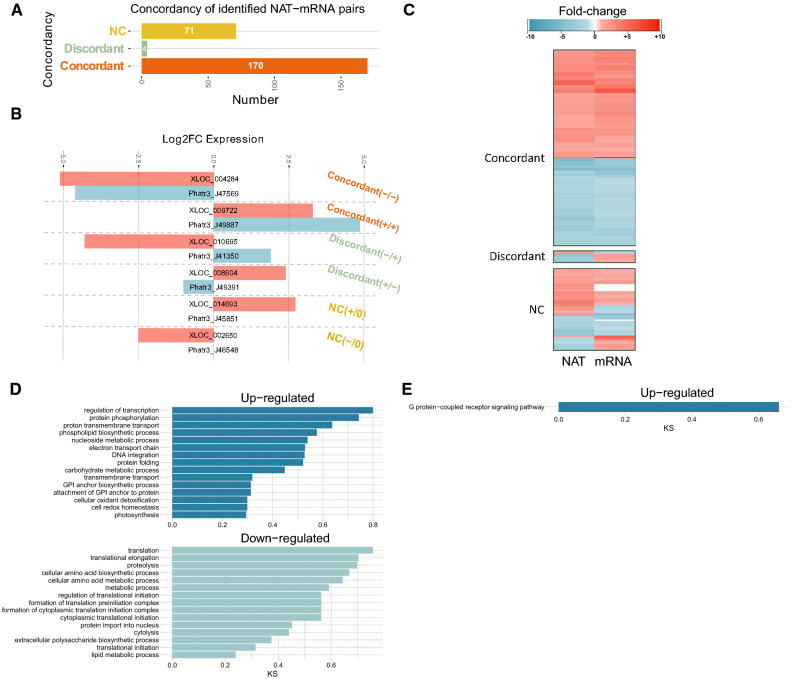
Characterization of the NAT-mRNA pairs detected in the Oval vs. the Fusiform pairwise comparison (OF). (**A**) Horizontal histogram showing the distribution of DE NAT-mRNA pairs according to the concordancy of the NAT and the corresponding cognate mRNA transcript. NC, low/no correlation (R^2^ ≤ 0.6) between NAT and its cognate mRNA. Discordant, NATs and their cognate mRNAs are DE with the opposite trend (one is upregulated while the other is downregulated), and Concordant, NATs and their cognate mRNAs are DE expressed with the same trend (negative or positive correlation), (**B**) fold expression change of two concordant, two discordant NAT-mRNA pairs, and one NC NAT-mRNA. (**C**) Heatmaps of log2 fold expression changes under morphotype change of the NAT-mRNA pairs (with positive and negative correlation) identified in *P. tricornutum* (R^2^ ≥ 0.6, fold change ≥ 2 or ≤ 1/2, and Adjusted *p* value < 0.05). Biological Process GO enrichment analysis of (**D**) concordant and (**E**) discordant NAT-mRNA pairs. Figures were made using the “Wes Anderson” color palette “Zissou1” in R (https://github.com/karthik/wesanderson).

In order to detect putative expression correlations between sense-antisense NATs and overlapping gene models forming NAT (-mRNA) pairs, we calculated the Pearson correlation coefficients (PCCs) of all the DE NATs versus their overlapping (> 50 nt) cognate protein coding genes (mRNAs). Of the 609 DE NATs detected in the OF pairwise comparison, 502 overlapped with a mRNA and 245 NATs overlapped with a DE mRNA in the same OF pairwise comparison (Supplementary Fig. S5). Amongst these, 97 correspond to a head-to-head overlap between the NAT and its cognate mRNA, 62 to a tail-to-tail overlap and 86 to a fully overlapped (Supplementary Fig. S5). We defined DE NATs-mRNA pairs if their expression responded to the following pattern: fold change ≥ 2, or fold change ≤ 0.5 and PCC r^2^ ≥ 0.6 (Supplementary Fig. S5). We then further defined concordant NAT-mRNA pairs as those in which the expression levels of both transcripts changed in the same direction between morphotypes and for discordant NAT pairs, the expression levels of the two transcripts should change in opposite directions. Between the TF pairwise comparison, only 15 DE NATs paired with a DE mRNA (Supplementary Fig. S5; Supplementary Table S4) and between the OT, 429 DE NATs paired with a DE mRNA (Supplementary Fig. S5; Supplementary Table S4). In the OF pairwise comparison, which represents the control fusiform form vs. the oval form, amongst the 245 DE NAT-mRNA pairs, 71 showed no expression correlation, while 170 correlated positively (concordant NAT-mRNA pairs) and 4 correlated negatively (discordant NAT-mRNAs pairs) (Fig. 4A–C; Supplementary Fig. S5; Supplementary Table S4).

### Insights into the putative regulatory functions of the DE NAT-mRNA pairs

We performed a search on the available gene ontology (GO) terms of the annotated transcripts with a correlated NAT (NAT-mRNA pairs with positive and negative correlation). Amongst the most abundant up-regulated GO annotation categories in the OF pairwise comparison were genes involved in the regulation of transcription, namely a chloroplast located protein with DNA binding activity (Phatr3_J47905), protein phosphorylation (Phatr3_J44344), proton transmembrane transport and phospholipid biosynthetic processes (Fig. 4D,E; Supplementary Table S5). Two phosphate transporter genes (Phatr3_J23830 and Phatr3_J40433) also formed a concordant NAT-mRNA pair up-regulated in the oval morphotype (OF). Down-regulated GO categories in the OF pairwise comparison included a big fraction of genes encoding membrane located proteins (Supplementary Table S5) and genes involved in translation and translation elongation.

### Comparison between DE genes under hyposaline conditions with those associated with the oval morphotype

**Figure 5:**
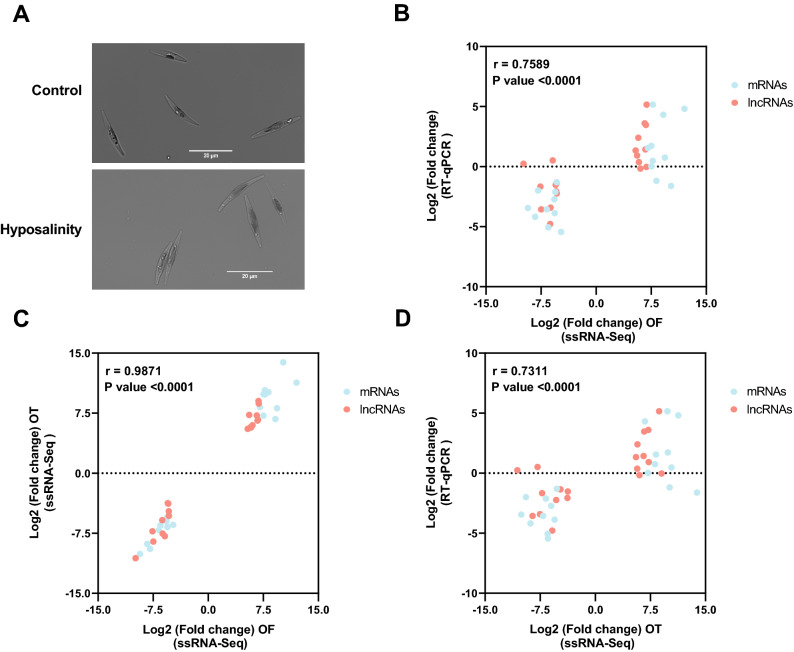
Analysis of the hyposaline effect compared to the impact of the morphotype in Pt1. (**A**) Culture of Pt1 at normal vs hyposalinity.After more than a year under hyposaline conditions, no shift in morphology was observed when compared to the control (100% seawater). (**B**) RT-qPCR vs RNA-Seq correlation of the top 40 DE selected genes in the OF pairwise comparison (Pt1 fusiform under hyposalinity conditions vs OF) r = 0.7589, *P* value < 0.0001. (**C**) Correlation of the expression profile of the 40 most DE selected genes in the OF with their expression profile in the OT, they show an almost perfect correlation (Pearson, r = 0.9871, p < 0.0001). (**D**) RT-qPCR vs RNA-Seq correlation of the 40 DE selected genes (fusiform under low salinity stress vs OT), Pearson correlation, r = 0.7309, *P* value < 0.0001. A 2^−ΔΔCt^ method was used for the normalization of the qPCR then a transformation was made to obtain the log2FoldChange. Correlation were performed using GraphPad Prism 9.4.1 analysis tool (Pearson correlation, *P* value Two-tailed, confidence interval 95%).

The original transcriptomes of the oval morphotype used in this study originated from hyposaline culture conditions (10% seawater), as opposed to the two others (fusiform and triradiate) that were cultivated under normal salinity (100% seawater) conditions^33^. For this reason, a salinity response cannot be excluded from the interpretation of the results, and downstream analysis, besides the morphotype difference. Taking this into consideration, we sought to investigate the salinity vs morphotype factor by studying the expression patterns of the top 20 differentially expressed genes (10 up and 10 down) of both non-coding and coding transcripts (lincRNAs and mRNAs, respectively) between the oval and the fusiform morphotypes (OF), obtained from the RNA-Seq data^33^. To do this we submitted the Pt1 line (which is > 99% fusiform in liquid culture) to similar hyposaline conditions which were used to maintain the Pt3 oval lines in Ovide and collaborators^33^. Both Pt1 and Pt3 belong to the same genotype (group A)^28,31^, which minimizes the molecular bias of analyzing different accessions. In our work, the Pt1 population kept the fusiform morphotype and did not transition to the oval morphotype as was previously reported^32^, even after prolonged hyposaline culture conditions (Fig. 5A).

We correlated the log2Fold change of the 40 selected genes from the RNA-seq with the RT-qPCR log2Fold change of the same genes in our hyposaline condition without changes in morphotype. Our results show a significant correlation (r = 0.76, *p* value < 0.0001) between the DE from the RNA-Seq datasets originating from the transcriptome of the oval morphotype vs fusiform (OF) in Ovide and collaborators^33^ and our RT-qPCR analysis of the fusiform morphotype under hyposaline conditions (Fig. 5B; Supplementary Fig. S6). Out of the 40 genes tested by RT-qPCR, 72.5% (29/40) (i.e., 70% (14/20) lincRNAs and 75% (15/20) protein coding genes top DE of the OF pairwise comparison) gave the same expression trend (up or downregulated) under hyposalinity stress as that observed in the oval morphotype transcriptome (Fig. 5B; Supplementary Fig. S6).

We then compared the DE transcriptomic data of these 40 genes (20 mRNAs and 20 lincRNAs) between the OF and the OT pairwise comparison and obtained an extremely high correlation (r = 0.99, *p* value < 0.0001) (Fig. 5C). A significant correlation (r = 0.73, *p* value < 0.0001) was also obtained between the RNA-Seq datasets of the DE 40 genes in the OT pairwise comparison with the RT-qPCR results obtained with a hyposaline culture (Fig. 5D; Supplementary Table S6).

## Discussion

Several studies across eukaryotes have shown the occurrence and functional relevance of lncRNAs in stress responses, cell identity and development^10,12,17,21^. Regarding unicellular eukaryotes such as diatoms and yeast, lncRNAs have been shown to be involved in nutrient stress responses, namely phosphate depletion^21–23^. Cell identity and development associated lncRNAs have been largely identified in multicellular organisms, revealing a restricted and highly cell-type specific expression of lncRNAs in different tissues^35^. Nonetheless, several studies made in yeast have been able to demonstrate the involvement of lncRNAs in the regulation of developmental processes in this single-cell organism^21^. For instances*, ICR1* and *PWR* are two related lincRNAs which have been shown to regulate phenotypic transitions in *Saccharomices cerevisae*, namely cell–cell adhesion during filament formation^36^, and *SUT169* is a NAT involved in sporulation regulation, also in the yeast *S. cerevisae*^37^.

*P. tricornutum* is the only diatom that has been shown to occur in three distinct morphotypes (fusiform, triradiate and oval) but the molecular underpinnings regulating the shift between the different morphotypes are unknown. It is therefore an interesting single-celled organism to investigate the involvement of lncRNAs in morphotype (or cell) identity regulation. The two strains that were under study in this work were Pt3, which is a subclonal culture of Pt2 collected off Plymouth UK (both are known as the Plymouth strain) able to grow in freshwater media^28^ and Pt1, collected off Blackpool, UK. Strain Pt3 has been reported to contain a majority of oval morphotype cells when cultured in seawater^28^. However, according to Ovide and collaborators, in order to maintain the oval morphotype it is necessary to keep the culture in hyposaline conditions^33^. This fact introduces a second variable which biases the analysis of the comparative studies between the three morphotypes in this Pt3 strain since, not only the morphotype is distinct but so is the culture medium. In this work we sought to investigate the differentially expressed (DE) lncRNAs between different morphotypes by doing pairwise comparisons of the three morphotypes. The idea behind this approach was to enable the identification of lncRNAs expressed in a specific morphotype and not the others, providing insights into putative regulatory roles in morphotype identity in *P. tricornutum*. We also deemed relevant to evaluate the salinity factor in the transcriptomic differences detected in the seemingly most distinct morphotype, which is the oval morphotype.

LncRNAs are recognized as being expressed several folds lower than mRNAs originating from the same tissue, cell type or stress condition, being a cross-kingdom feature that has been reported in several different systems including mammalian cells^7,38–40^, plants^41–43^ and yeast^44^. However, they are also recognized as being more specifically expressed than protein coding genes^12,45,46^. Both these features were previously reported for lncRNAs detected in *P. tricornutum* submitted to phosphate fluctuations^22,23^. In the present study, we found a similar trend. LncRNAs were not only expressed at lower levels than mRNAs across the three morphotypes of the Pt3 strain (Fig. 1) but were also more specifically expressed, lncRNAs clustering according to a morphotype, clearly separating the three morphotypes, including the fusiform from the triradiate that did not occur in the clustering of DE mRNAs (Fig. 3).

Between the three pairwise comparisons of differentially expressed (DE) lncRNAs, the comparison displaying the fewest DE lncRNAs was that between the triradiate and the fusiform (TF) morphotypes, for both lincRNAs and NATs (Fig. 2). Similar results were obtained for protein coding genes^33^. Furthermore, 85–87% of the DE detected in the OF pairwise comparison were also detected in the OT pairwise comparison. Taken together, the oval morphotype seems to clearly separate itself from the other two (fusiform and triradiate) in terms of the numbers of DE in both long noncoding (Fig. 3) and protein coding genes^33^. Nonetheless, in terms of morphology all three forms are very distinct to each other but, this does not translate directly into the amplitude of DE genes between the three pairwise comparisons (Fig. 2). Indeed, the triradiate represents a very distinct morphotype to the fusiform and yet the DE differences between the two are significantly lower than the DE expressed differences between the two others pairwise comparisons (Fig. 2; Supplementary Table S3). Furthermore, the oval morphotype has been typically associated with stress conditions such as nutrient, temperature, salinity stress and solid media^29,32^. Considering the fact that the oval transcriptomes originate from low salinity cultures, as opposed to the two other morphotypes (fusiform and triradiate) which were cultivated in 100% seawater^33^, led us to evaluate the effect of a hyposaline stress in the fusiform morphotype using the reference strain (Pt1) which is ~ 99% fusiform under normal liquid culture conditions. For this we exposed Pt1 cultures to prolonged hyposaline conditions (and other stresses such as prolonged darkness, lack of agitation, lack of environmental renewal and a combination of those factors over several months) and no overall change to the oval morphotype was observed. Among the 40 genes analyzed, ~ 90% of the downregulated genes from the OF pairwise comparison originating from the RNA-Seq data displayed the same tendencies by RT-qPCR and the same applies for ~ 60% of the upregulated genes (Fig. 5C; Supplementary Fig. S6). This suggests that the DE transcriptome of the oval morphotype would be, to a large extent, a hyposalinity response transcriptome overshadowing the molecular differences exclusively related to the morphotype. Furthermore, the DE of these top 40 genes was highly correlated in both OF and OT pairwise comparisons (Fig. 5C), suggesting that the expression of these genes was not correlated to the morphotype but rather to the salinity of the culture medium (normal seawater for the fusiform and triradiate vs hyposaline in the oval morphotypes). Nonetheless, we suggest that amongst the DE genes which do change between the two pairwise comparisons (OF and OT) (Fig. 2D,F), might be genes specifically associated to the fusiform or the triradiate morphotype. Also, amongst the genes tested by RT-qPCR that were highly DE in the OF pairwise comparison and did not respond to the hyposalinity culture, are potential genes specifically associated with the oval morphotype (Supplementary Fig. S6). LncRNAs falling in these categories (morphotype specifically associated) could be potential regulators of morphotype identity, or morphotype shift and maintenance, in *P. tricornutum*.

Many lncRNAs have been described to exert their regulatory roles by associating with chromatin modifying complexes, namely with the evolutionary conserved polycomb repressive complex 2 (PRC2), which mediates gene silencing during cell differentiation and development in both animals and plants^47–50^. Interestingly, a recent report in *P. tricornutum* showed that the knockout of a component of PRC2 (enhancer of zeste E(z)) lead to a decrease in the repressive H3K27me3 chromatin marks which could be correlated to morphotype shift indicating an involvement of PRC2 in the regulation of morphotype identity in *P. tricornutum*^51^. Taken together, we can suggest that morphotype identity regulation in *P. tricornutum* could involve specific lncRNAs that function similarly to those identified in plants and animals, targeting PRC2 to specific genomic loci which, through the deposition of repressive chromatin marks, determine morphotype identity. Potential morphotype regulatory lncRNAs candidates could be found amongst the DE lncRNAs detected between the triradiate and fusiform morphotypes, namely 4 lincRNAs and 5 NATs that are exclusively DE between these two morphotypes (Fig. 2D,F; Supplementary Table S3). Interestingly the 4 lincRNAs exclusively DE in the TF pairwise comparison (XLOC_002740, XLOC_010810, XLOC_003535, XLOC_011321) are significantly downregulated in the triradiate, and so are 2 of the 5 DE NATs (XLOC_002164, XLOC_004596) (Supplementary Table S3). This could suggest that the expression of these lncRNAs is needed to maintain the fusiform morphotype and that their expression needs to be downregulated in order for the shift to the triradiate morphotype to occur. On the other hand, 3 NATs specifically DE in the TF pairwise comparison are highly upregulated in the triradiate form (XLOC_015151, XLOC_002397, XLOC_000971) (Supplementary Table S3), suggesting that their expression is putatively related to the maintenance of this morphotype. In the case of the oval morphotype, when removing the salinity responsive DE lincRNAs, we could detect 4 lincRNAs which could be specifically associated to this morphotype (XLOC_002999, XLOC_006277, XLOC_007078, XLOC_007821; Supplemental Fig. S6).

In conclusion, with this work we show that in the marine diatom *P. tricornutum* DE lncRNAs provide a specific morphotype signature and, several lncRNAs specifically associated to a morphotype (fusiform, triradiate and oval) have been identified, which suggests a role for these noncoding transcripts in cell identity. Functional studies by a knockout approach currently underway will be extremely useful in elucidating the roles of these candidate lncRNAs in morphotype identity and salt stress responses in *P. tricornutum*.

## Supplementary Information

Supplementary Information 1.

Supplementary Information 2.

Supplementary Information 3.

Supplementary Information 4.

Supplementary Information 5.

Supplementary Information 6.

## Data Availability

The sequencing raw data used in this study was retrieved from the European Nucleotide Archive ENA using the accession number PRJEB26173.
