# Supplementary material for: Differential expression patterns of long noncoding RNAs in a pleiomorphic diatom and relation to hyposalinity
Source: Sci Rep. 2023 Feb 10;13:2440. doi: 10.1038/s41598-023-29489-w (PMC9918465; doi:10.1038/s41598-023-29489-w)
Supplement: Supplementary file 1 — Supplementary Information 1. [file 41598_2023_29489_MOESM1_ESM.pdf]

Figure S1

A.

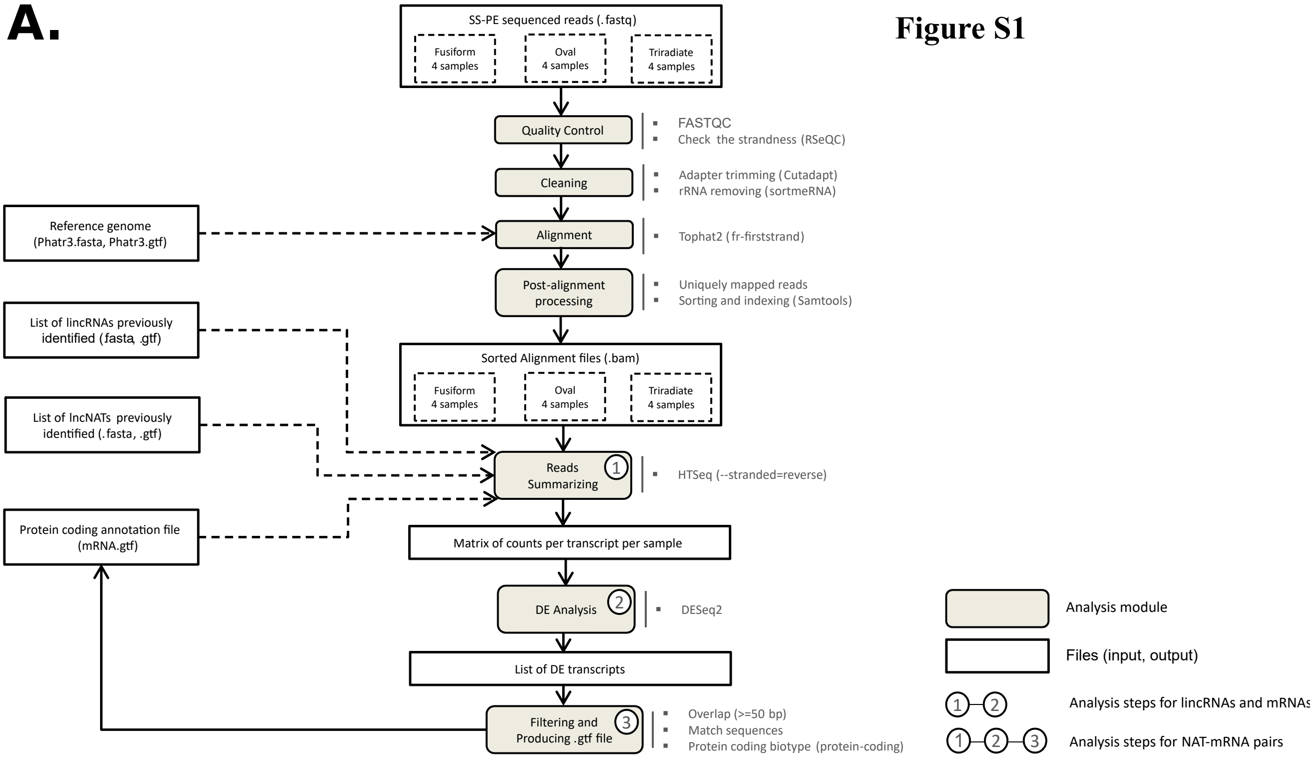

B.

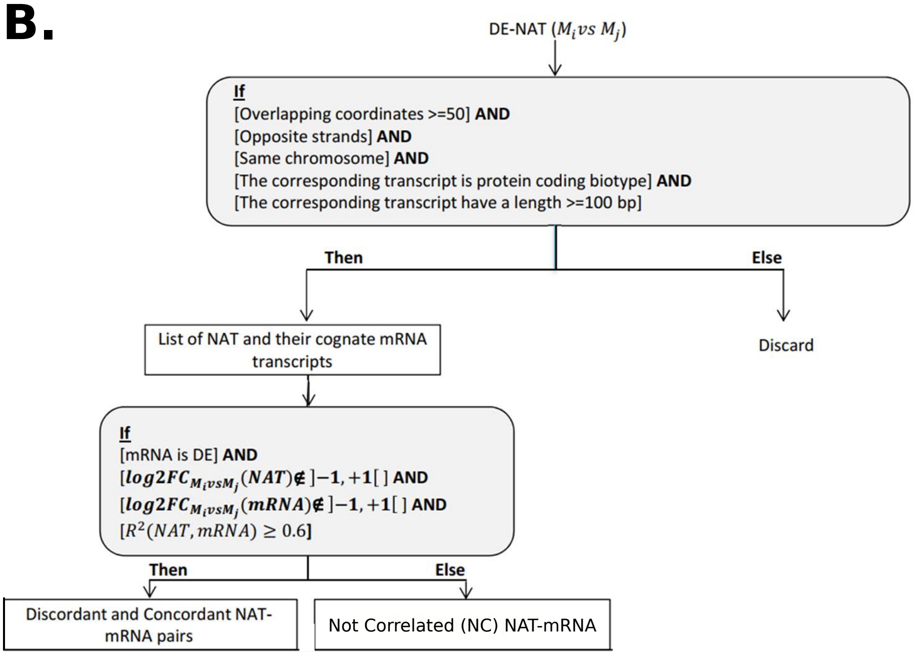

Figure S1. Pipeline overview of the workflow used for the identification and the analysis of lincRNAs, NATs and Protein-coding genes. (A) Differential expression analysis pipeline: Stranded RNA-seq libraries of the three morphotypes with four replicates for each were downloaded from the SRA portal. The FASTQ files serve as input for RNA-seq DE analysis. After preprocessing of the reads including QC, checking the strandedness of the libraries, adapter trimming, and ribosomal RNA rRNA removing, the reads were aligned to the reference *Phaeodactylum tricornutum* genome (Phatr3) using Tophat2 with the option *--library-type fr-firststrand.* The resulting files were then sorted and indexed using samtools before quantifying the reads using HTSeq-count (module 1) including the strandedness information *--stranded=reverse*. Quantification tools generated a count matrix per transcript (rows) per sample (columns) which was subsequently filtered and normalized using the median of ratio method of DESeq2. The resulting matrix was then used for Differential Expression DE analysis (module 2) for each pairwise comparison (Oval versus Fusiform, Oval vs Triradiate, and Triradiate vs Fusiform). The DE was done using DESeq2 package with the Wald test, and the Benjamini-Hochberg BH method was used to correct for multiple testing. The route 1 > 2 was used for lincRNAs and mRNA DE analysis, while the route 1 > 2 > 3 was used for NAT-mRNA pairs analysis. The module 3 took the list of DE NATs in input, and by using a series of post-processing and filtering, the module produced an annotation GTF file containing all the mRNA (protein-coding) transcripts overlapping with a DE NAT (min overlap >= 50bp). The output file (mRNA.gtf) was then used to detect DE mRNA transcripts. (B) An analysis pipeline was used for the detection of NAT-mRNA pairs for each pairwise comparison. The pipeline outputted NAT-mRNA pairs and classified them as concordant, discordant, and NC.

Figure S2

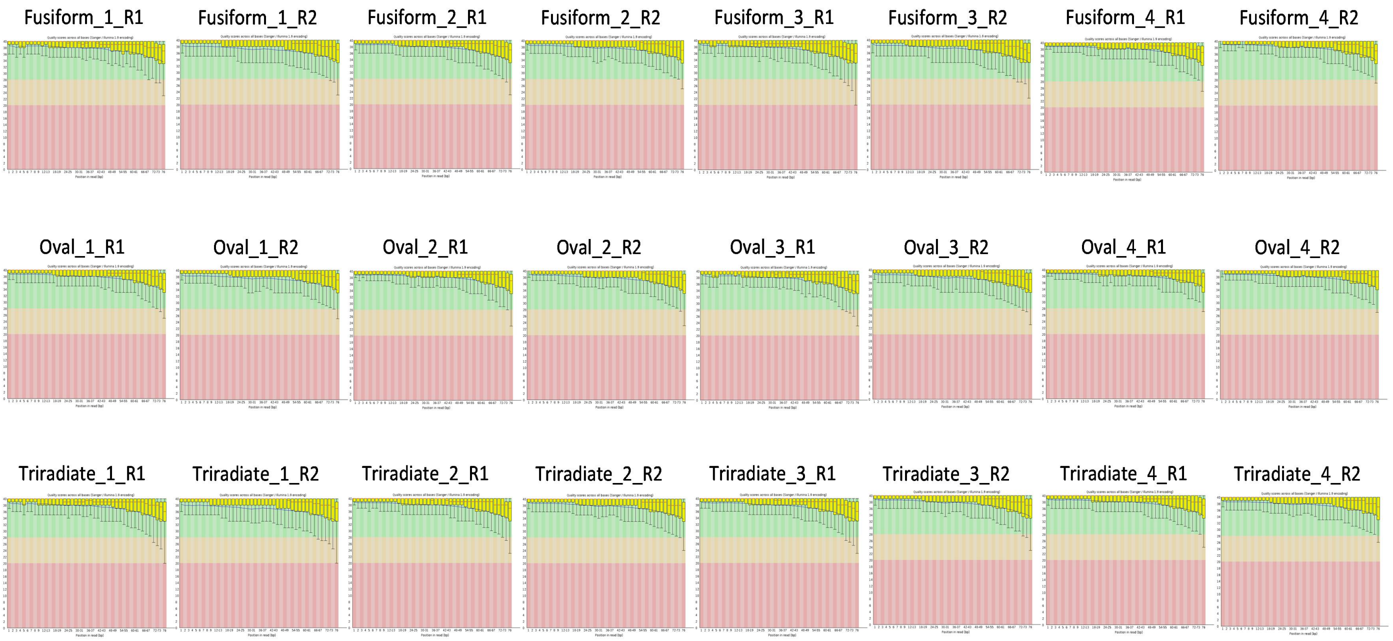

Figure S2. **Quality check of the reads.** The per base sequence quality report (FASTQC) for the 4 biological replicates of the respective enriched cultures for either the fusiform, triradiate or oval morphotype.

Figure S3

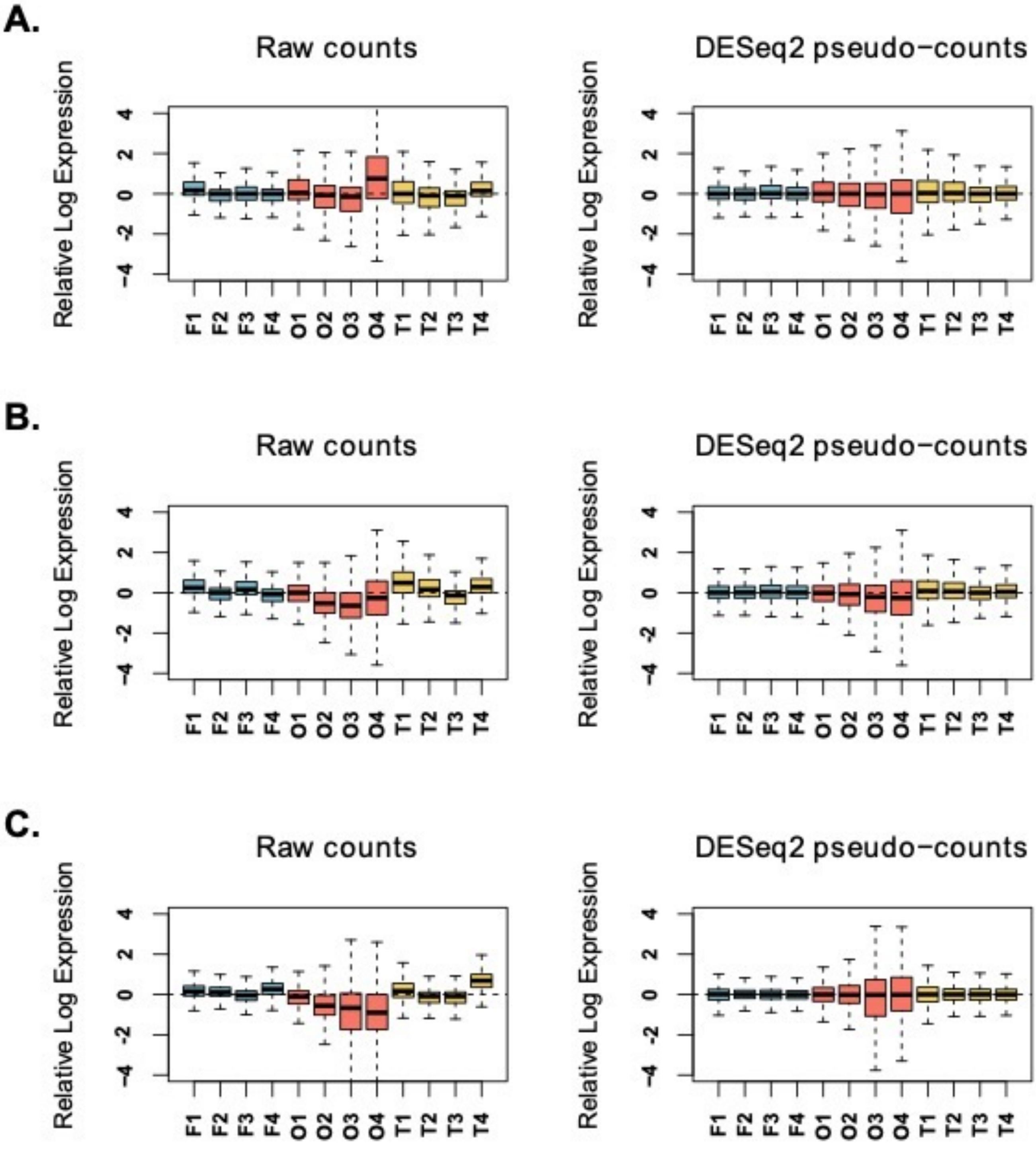

Figure S3. QC of the DESeq2 internal normalization. Distribution of raw counts per sample (left) and DESeq pseudo-normalized counts (right) using the summarized matrix of counts obtained for (A) lincRNA (B) NAT, and (C) mRNA transcripts. The normalized counts were used in all downstream analyses. Boxplots were plotted using the plotRLE function of the EDASeq package.

Figure S4

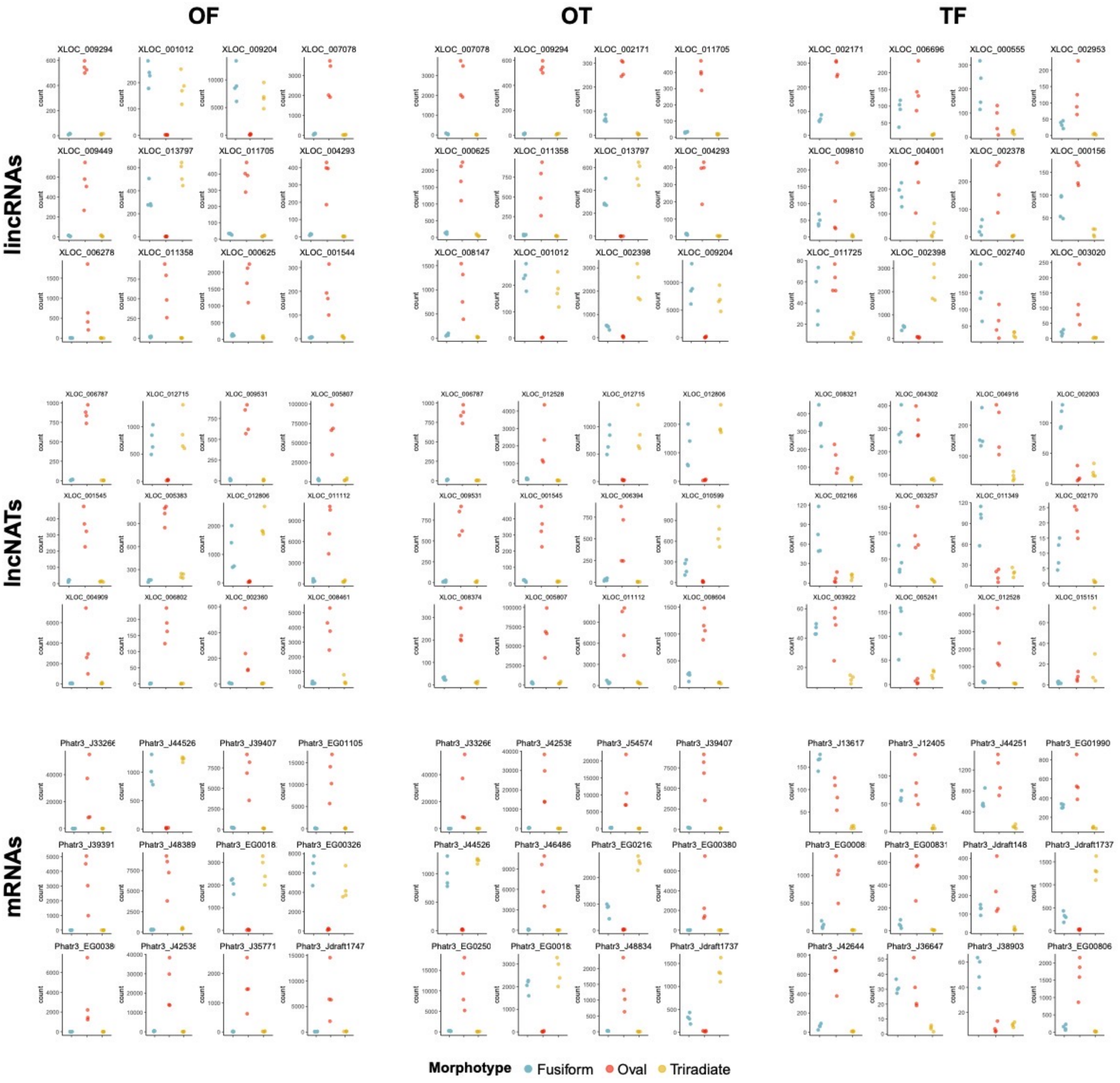

Figure S4. Top 12 DE linc for OF (and the other two pairwise comparisons). The output of the *plotCounts* function of the DESeq2 package showing the differences in expression between the three morphotypes of the top12 most differentially expressed transcripts (rows) per pairwise comparison (columns).

Figure S5

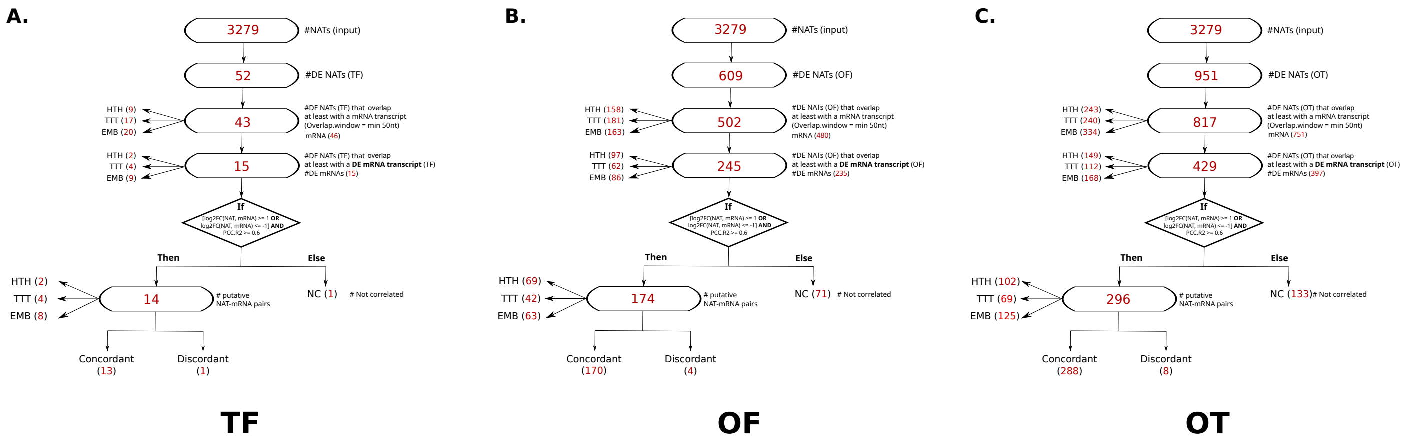

Figure S5. Flowchart depicting the number of detected NATs per step: (A) OF (B) OT (C) TF. Detailed schematic diagram of the bioinformatic pipeline for the identification of NAT-mRNA pairs (see Figure S1. B) for each pairwise comparison (A) TF, (B) OF, and (C) OT. The transcripts were filtered in each step to identify non-coding antisense transcripts. The number of candidate NATs retained after each step is also reported.

Figure S6

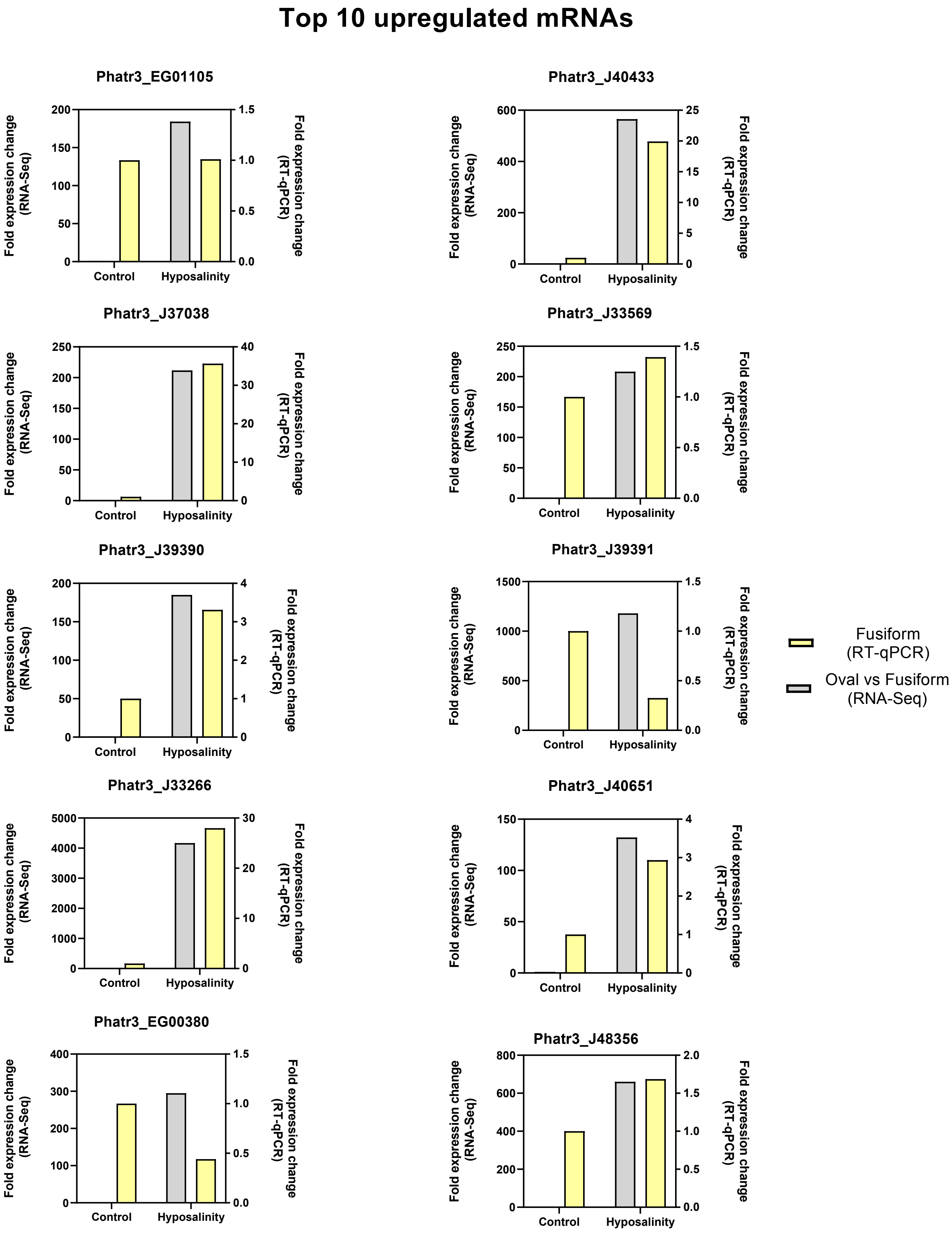
Top 10 upregulated mRNAs
Phatr3_EG01105
Phatr3_J40433
Phatr3_J37038
Phatr3_J33569
Phatr3_J39390
Phatr3_J39391
Phatr3_J33266
Phatr3_J40651
Phatr3_EG00380
Phatr3_J48356
Fold expression change (RNA-Seq)
Fold expression change (RT-qPCR)
Control
Hyposalinity
Fusiform (RT-qPCR)
Oval vs Fusiform (RNA-Seq)

Figure S6

Top 10 downregulated lncRNAs

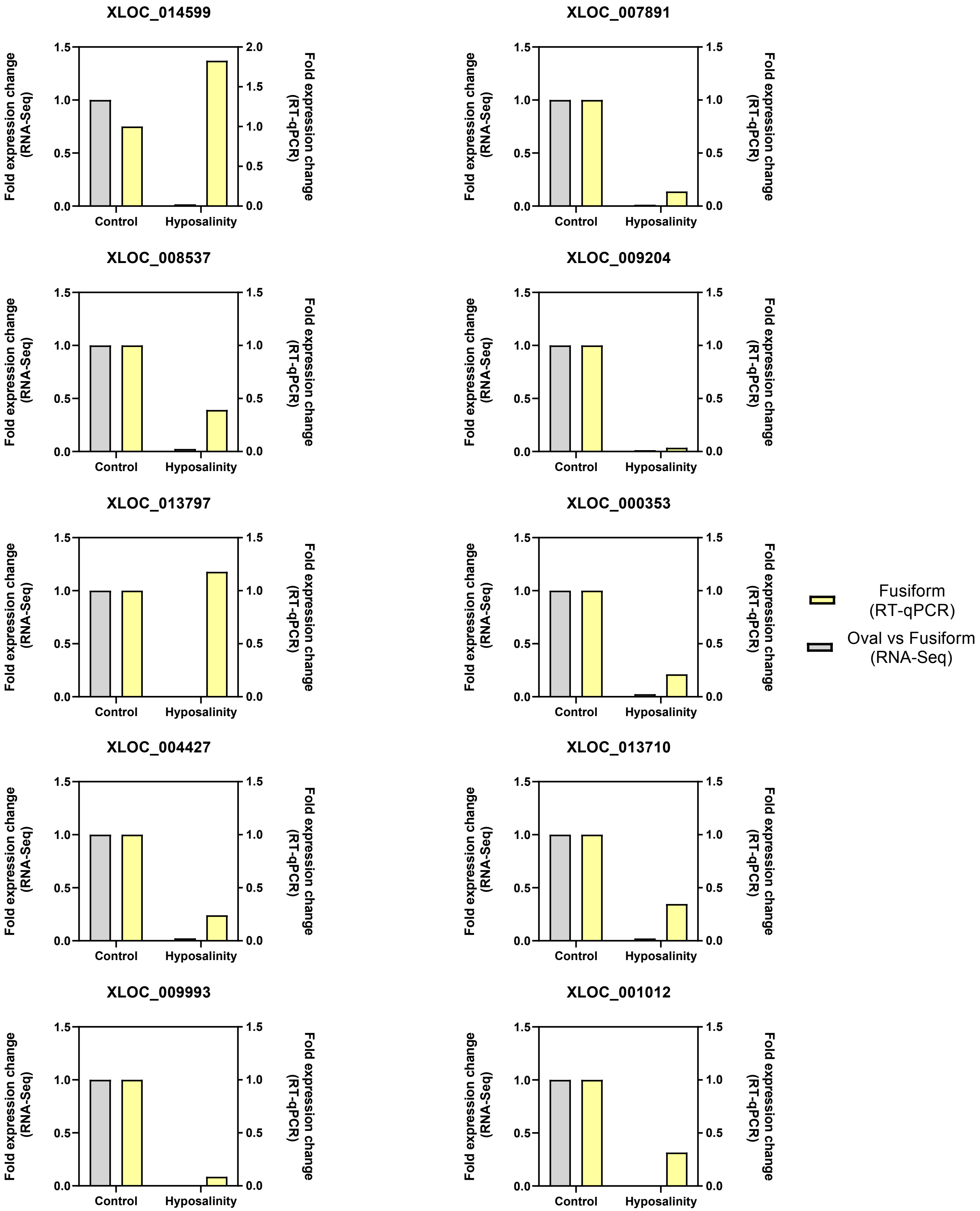

Figure S6

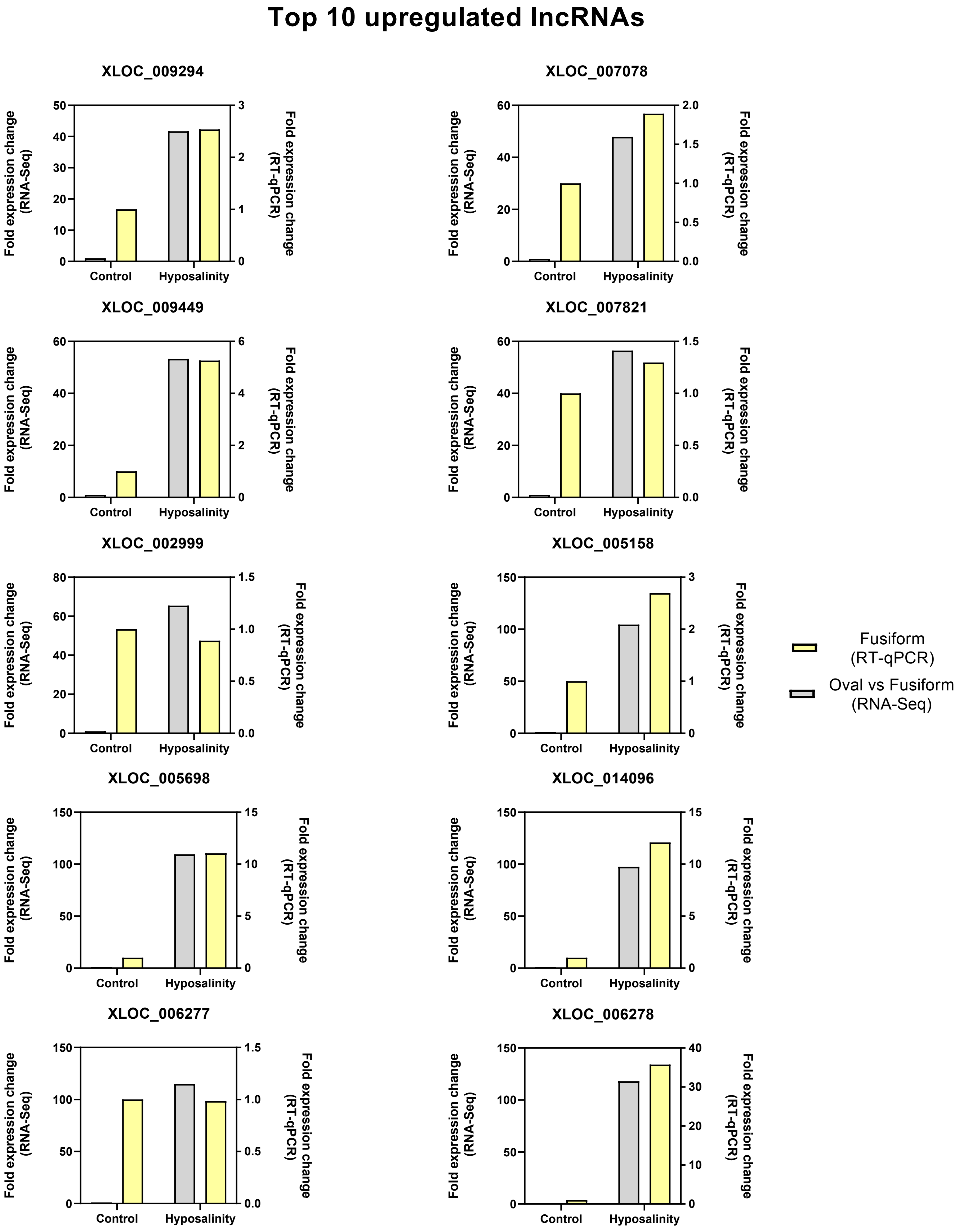
Top 10 upregulated lncRNAs
XLOC_009294
XLOC_007078
XLOC_009449
XLOC_007821
XLOC_002999
XLOC_005158
XLOC_005698
XLOC_014096
XLOC_006277
XLOC_006278
Fold expression change (RNA-Seq)
Fold expression change (RT-qPCR)
Control
Hyposalinity
Fusiform (RT-qPCR)
Oval vs Fusiform (RNA-Seq)

Figure S6

Top 10 downregulated mRNAs

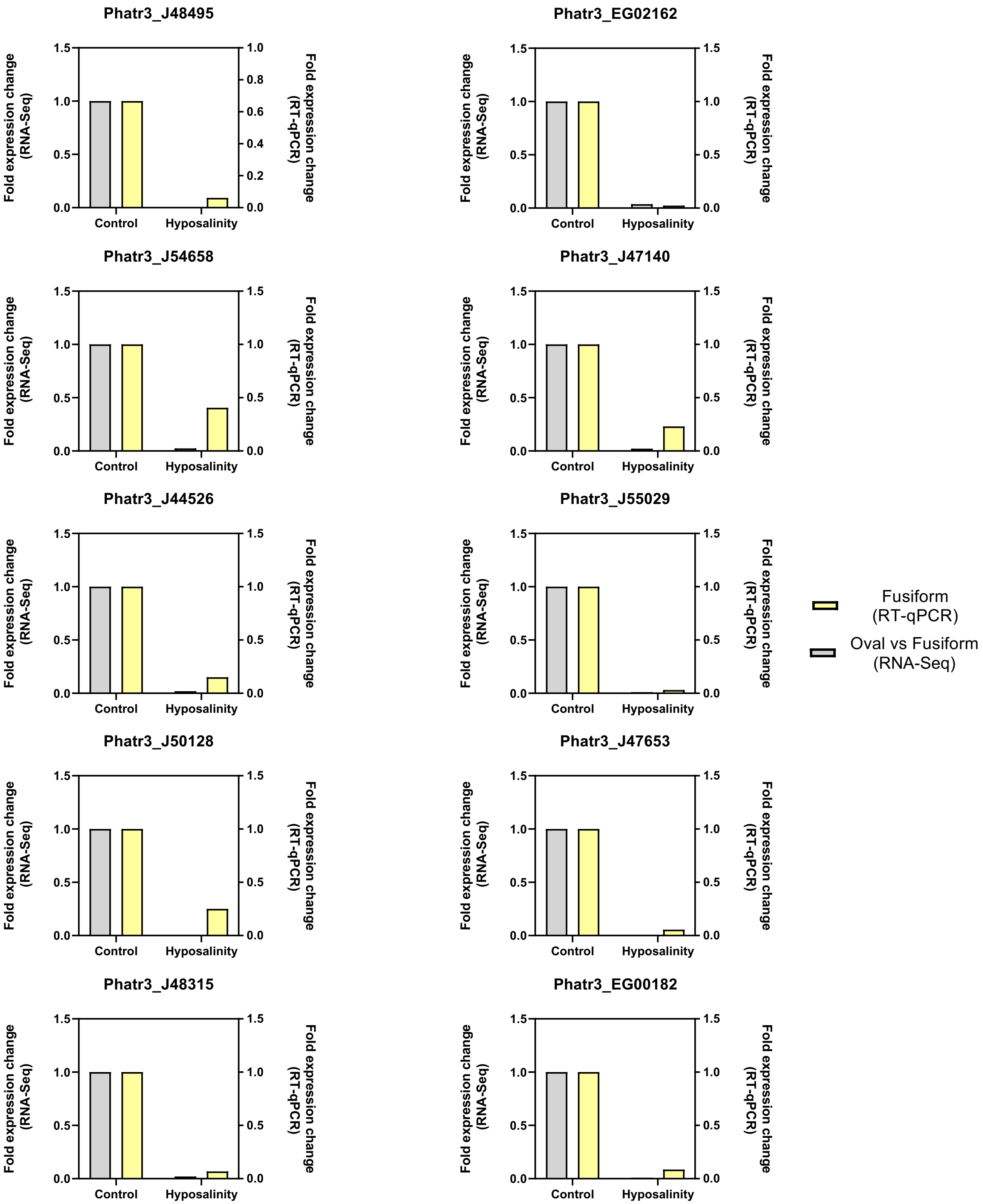

Figure S6. Comparison of the top 40 DE genes between RNA-seq (OF) and RT-qPCR (Fusiform) RT-qPCR analysis of the 40 most differentially expressed genes (top 10 up/down mRNAs and lncRNAs) based on the RNA-seq data from Ovide et al. (2018) (OF comparison). The values compared are the RNAseq fold change (yellow) and RT-qPCR fold change (grey) calculated and normalised by the 2^-ΔΔCt method (as described in Material & Methods). Results are the mean of three biological replicates for each qPCR.
