# Supplementary material for: Differential expression patterns of long noncoding RNAs in a pleiomorphic diatom and relation to hyposalinity
Source: Sci Rep. 2023 Feb 10;13:2440. doi: 10.1038/s41598-023-29489-w (PMC9918465; doi:10.1038/s41598-023-29489-w)
Supplement: Supplementary file 2 — Supplementary Information 2. [file 41598_2023_29489_MOESM2_ESM.pdf]

Table S1. QC and Alignment. The strandedness and metric of mapping from Tophat2 for the 4 biological replicates of the 3 morphotypes: Fusiform, Oval, and Triradiate.
[1] Fraction of reads explained by "1+-, 1-+, 2++, 2--" as obtained by *infer_experiment.py* script (RSeQC package) meaning the read1 (paired-end data) is located on the opposite strand as the transcript. [2] Concordant pair alignment rate as returned by Tophat2

**Sample**	SRA sample name	Strandedness[1]	#Input reads	#cleaned reads	Alignment rate[2] (%)	**Unmapped reads (%)**
Fusiform (F1)	ERR3285011	0.93	23117449	22315103	85.3	8.9
Fusiform (F2)	ERR3285012	0.95	15298228	14762829	87.5	7.7
Fusiform (F3)	ERR3285013	0.91	17634774	17170250	87.1	8.0
Fusiform (F4)	ERR3285014	0.96	17925360	17687742	88.8	7.3
Oval (O1)	ERR3285015	0.94	18073097	17370215	85.9	8.2
Oval (O2)	ERR3285016	0.96	17996250	17453353	88.1	7.1
Oval (O3)	ERR3285017	0.95	16397447	15937826	88.0	7.1
Oval (O4)	ERR3285018	0.93	23285568	22969577	87.4	7.4
Triradiate (T1)	ERR3285019	0.90	22445606	21377419	87.0	8.1
Triradiate (T2)	ERR3285020	0.91	14314071	14033279	87.3	7.6
Triradiate (T3)	ERR3285021	0.95	18289429	17912397	87.6	7.2
Triradiate (T4)	ERR3285022	0.95	23393382	22955812	88.4	6.9

Table S2. RT-qPCR Primers. List of primers used for the qPCR experiment. Primers were designed as described in the method.

ID - Phatr3	Seq- Fwd	Seq-Rev
**Phatr3_J29812**	GCCTTACACCGCCGTATTCT	TCAATGACAAGGGCAGCGAT
**Phatr3_J19761**	CCCGAATTCAACCGAAAGGC	ATGGCAGCCTTGAGAGAGC
**Phatr3_EG00182**	GCTACGGAGAGCCTGATGTC	CTTTTGTGGCCCTTTCCACG
**Phatr3_EG00380**	ATTCACCACGGCAACGGTAT	CCCGTTCCCGGATAAAACCA
**Phatr3_EG01105**	TGAACTTCAATACATTCGATGCG	GACCGTTTCGTTGCTGTACC
**Phatr3_EG02162**	TGGTTTGTGTTTCCCGCACT	TTTGTGGAACCACCCGGTAA
**Phatr3_J33266**	TTTCACCTGTCTGGCTGGTC	ACAATCGCCAAGTACCCGTT
**Phatr3_J33569**	CTACAGCAACCGACATGGGA	AAGGTGGGCAAGTAGTTCCG
**Phatr3_J37038**	TGGAAACCAGGAGATGTCGC	ACTCCAAGCTCACGCTTCTC
**Phatr3_J39390**	CGCTCAGAGCAAAGTAGCCT	GGTACACGCCGACTCTTGAA
**Phatr3_J39391**	AGTTTTCGACGGCGATACGA	GTCCCGCAGTTTTGGGGATA
**Phatr3_J40433**	ATGTCTGCCCTTGTCTCTGC	CACTCAACGGAACTTGACGC
**Phatr3_J40651**	TCCTCGCAATATTCCCGTCG	TGGTTGGATTCGCCATTGGA
**Phatr3_J44526**	GTACCTTCGGAGCATACGCA	CAGACGCCATCTCGTTGTTTG
**Phatr3_J47140**	GGCACGTGGTCTACTGAGTC	CTGGTTCCGGCACAATCTCT
**Phatr3_J47653**	GCCCAACACGATTGGTAGGA	GGAAGGCCCATTGTGACGTA
**Phatr3_J48315**	GACATCGCTTCAACTTGCCC	GTACAAACGCCGGCAACAGT
**Phatr3_J48356**	GCTCCCACGGAGTAGTGAAC	CGAGTTGGGGATGTACGTGG
**Phatr3_J48495**	ATACTAATGAAGCCTGTGGGTC	TGACATCGTGCAGAGACACC
**Phatr3_J50128**	CAAGCTCCACACCTTTTGGC	TATTCTGGGAGTGACCCCGT
**Phatr3_J54658**	AGCACAATTCTCGCTGTGGA	CCATCCGTCTTGACTGCCTT
**Phatr3_J55029**	CTCCCGACAACTGGTATAGGC	GCACGTTCCAGAATAGAGTCCA
**XLOC_000353**	GATCGTCATCACCACCCTCC	TACTGCCAGTGAAACGCACA
**XLOC_001012**	GACAAGAGACTCCAGCCGAG	TTCGTATACCGCAGACGAGC
**XLOC_002999**	GCAACCCCTCCTTCGCTAAA	CTGGACGGGATATCGGATGG
**XLOC_004427**	GCCGTGAAGCCAATCAAGTG	TTGTTCTGGCCAACTCCCTC
**XLOC_005158**	TCCATACTTTCGCAGAGGCG	ACCACAAGGACGCAGGATTA
**XLOC_005698**	CGATTGATGGTCCGGCTAGA	GCAATAGCATTACTCTTCTTCTTCA
**XLOC_006277**	TCGACCTGCAAGATCGCTTC	AGCTGTACGAATGGGTTGCT
**XLOC_006278**	GCAAAGCCTTCTTCCCCAGA	TCCTCGGTAAACTCGTTAGGC
**XLOC_007078**	GCCAGCACCGAGAATGTTTG	GGACATTCAGACCGACCGTA
**XLOC_007821**	CGGCAAAGTTCCATCAGCAA	TGCAATTGACGGTGGTGAGA
**XLOC_007891**	GGTCACGTTGCCGATTTGTT	CGTTCTGTCCCCTTTTGGGA
**XLOC_008537**	CTCAGGCCAAGTTGGGAACA	TGCCAACATGGACCAATGGA
**XLOC_009204**	TGTCCCGTTTTGCATTGCTC	TGTCATGTTTTGGCCTCCGA
**XLOC_009294**	GGAGACTTACGACCGCGAAG	CAGCCATCATGTCATCGCCTA
**XLOC_009449**	TTCGCCGTCAAGTACGGTAT	CCAGGGTTCCAGCATCCATT
**XLOC_009993**	GACATCAGGCTCTCCGTAGC	ACACACCAAAACCGTCCCTT
**XLOC_013710**	GTTCGTGCCAGTAGTCAACAC	TCTGATTCGCTCTGTCACCG
**XLOC_013797**	ATGATCTGCCCGAACGTACA	ATCCTTTGGTCCCACAGACAC
**XLOC_014096**	AGACACCAGCGCAACATACA	GCCAAGCAATCCAGGAAAGG
**XLOC_014599**	TCATGCAAAGCAAGAACCATCA	GCACAATGGAGCCATACAAACT
